# E-Tongues/Noses Based on Conducting Polymers and Composite Materials: Expanding the Possibilities in Complex Analytical Sensing

**DOI:** 10.3390/s21154976

**Published:** 2021-07-22

**Authors:** Alfonso Sierra-Padilla, Juan José García-Guzmán, David López-Iglesias, José María Palacios-Santander, Laura Cubillana-Aguilera

**Affiliations:** 1 Institute of Research on Electron Microscopy and Materials (IMEYMAT), Department of Analytical Chemistry, Faculty of Sciences, Campus de Excelencia Internacional del Mar (CEIMAR), University of Cadiz, Campus Universitario de Puerto Real, Polígono del Río San Pedro S/N, 11510 Puerto Real, Cadiz, Spain; alfonso.sierra@uca.es (A.S.-P.); laura.cubillana@uca.es (L.C.-A.); 2 Instituto de Investigación e Innovación Biomédica de Cadiz (INiBICA), Hospital Universitario ‘Puerta del Mar’, Universidad de Cadiz, 11009 Cadiz, Cadiz, Spain; juan.garcia@inibica.es

**Keywords:** conducting polymers, electronic tongues, electronic noses, chemometric, discrimination, analysis

## Abstract

Conducting polymers (CPs) are extensively studied due to their high versatility and electrical properties, as well as their high environmental stability. Based on the above, their applications as electronic devices are promoted and constitute an interesting matter of research. This review summarizes their application in common electronic devices and their implementation in electronic tongues and noses systems (E-tongues and E-noses, respectively). The monitoring of diverse factors with these devices by multivariate calibration methods for different applications is also included. Lastly, a critical discussion about the enclosed analytical potential of several conducting polymer-based devices in electronic systems reported in literature will be offered.

## 1. Introduction

In the last decades, intrinsic conducting polymers (CPs) have attracted wide attention due to their excellent electrochemical characteristics, such as tailored electrical conductivity by electronic doping, high environmental stability, and biocompatibility. Their electrical and optical properties establish them as excellent candidates for chemical sensing applications targeting the detection/determination of diverse analytes of interest. In this sense, electrochemical sensors [1,2,3], gas sensing devices [4], and optical sensors [5,6] have been proposed as providing good analytical features in terms of figures of merit (e.g., sensitivity, limits of detection and quantitation, repeatability, reproducibility, linear range, and robustness). In addition, the modulation of instrumental conditions during the electrodeposition process [7], spin coating [8], or sequential solution polymerization technique [9,10] allows precise control over the thickness and morphology of the resulting conducting coating. This advantage is particularly important in the development of electrochemical and gas sensors [11,12,13].

**Figure 1:**
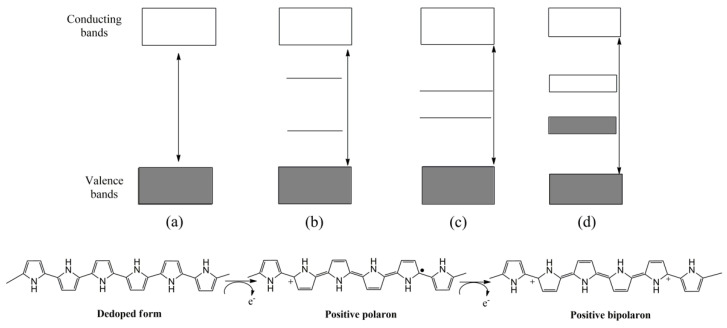
Schematic representation of p-doping process of PPy and structure of electronic bands in different electronic states: (**a**) de-doped, (**b**) polaron, (**c**) bipolaron, (**d**) bipolaron coupling.

In this regard, nanostructures can also be conveniently tailored, leading to analytical sensing improvements [11]. In addition to their morphology, their electrochemical properties can be customized by electronic doping (p or n) extensively reported in the bibliography [14,15]. As an illustrative example, the p-doping of PPy is shown in Figure 1. In the first stage, a radical cation is formed by the oxidation of the polymer, inducing a local deformation within the polymer chains, leading to the formation of intermediate electronic states between the conducting and valence band. Subsequently, the polymer can be re-oxidized, increasing the number of charge carriers [16].

This is the reason why the electrochemical characteristics of the conducting polymers, such as electrical conductivity, can be modulated by electronic doping. This characteristic feature allows the employment of conducting polymers in several applications previously mentioned, such as electrochemical transducers in sensor devices, electrochromism, solar cells, batteries and supercapacitors, among others, proving their versatility in comparison with other electrode materials. Furthermore, the entrapment of enzymes within the polymeric layer may increase the selectivity of the overall system. In this sense, several examples, including horseradish peroxidase [17], tyrosinase [18], and glucose oxidase [19] can be found in the literature.

**Figure 2:**
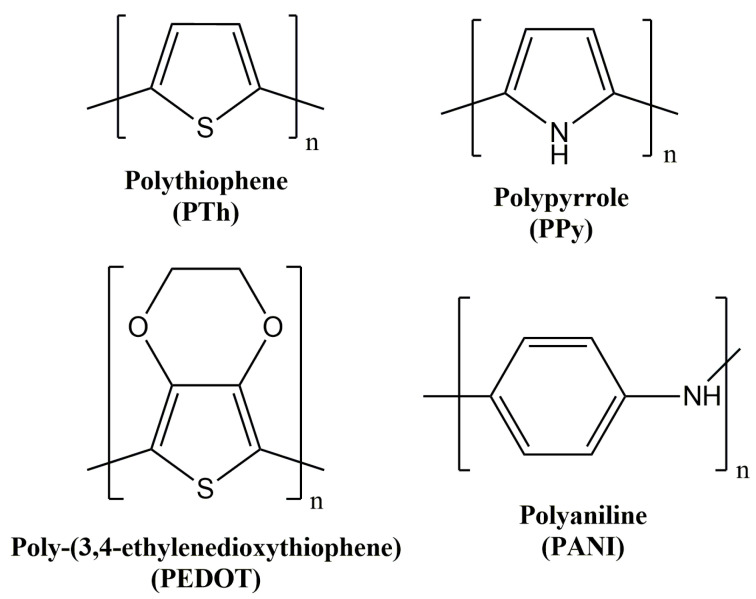
Chemical structure of the most relevant intrinsic conducting polymers.

Despite the high number of intrinsic conducting polymers present in the bibliography, great attention was focused on polythiophene and the corresponding derivatives, as well as on polyaniline and polypyrrole (Figure 2).

### 1.1. Polythiophene and Derivatives

Polythiophene (PTh) have several electroactive properties, such as the remarkable ability for doping/de-doping and high electrical conductivity, which makes them excellent choices for electrochromic supercapacitors and electrochemical applications [20]. Nonetheless, high potentials are required to oxidize the unsubstituted thiophene ring, which can induce the overoxidation of the resulting polymeric film. The inclusion of functional groups in the thiophene monomer leads to a significant reduction of the potentials required for the oxidation due to the presence of electron donor inductive effects [21,22]. Among all the polythiophene derivatives, poly-(3,4-ethylenedioxythiophene) (PEDOT) raised based on its low oxidation potential, which provides a wide oxidation potential window. Hence, the direct electrochemical sensing of different electroactive species can be performed using PEDOT-based electrochemical devices [23,24]. Moreover, the entrapment of biological species onto PEDOT films by several procedures, such as sinusoidal current [25], sinusoidal voltages [26], and drop casting [27] has been achieved.

### 1.2. Polyaniline

Polyaniline (PANI) is constituted by three ideal oxidation states: leucoemeraldine (reduced form), emeraldine (half-oxidized state), and pernigraniline (fully oxidized state) [28]. The reversible redox conversion between emeraldine base, non-conducting form, to emeraldine salt, conducting form, has been exploited in several electronic devices, e.g., gas sensors, supercapacitors, electrochromic devices, and electrochemical sensors [29,30,31,32]. Regarding electrochemical devices, the electroactivity of polyaniline film plays a relevant role. Low electroactivity of the polyaniline film at neutral and basic electrolytic media can be found, which can be ascribed to the deprotonation of emeraldine salt at higher pH. Polyaniline composites were developed and used for electrochemical sensing to avoid the deprotonation of the conducting polymer backbone. In this sense, self-doped polyaniline-based devices showed electroactivity at neutral media [33,34,35].

### 1.3. Polypyrrole

Polypyrrole (PPy) is a versatile conducting polymer, characterized by redox properties, high electrical conductivity, and reversible redox switching [36]. The redox conversion of neutral form (yellow) to oxidized form (black grey) is useful for electrochromic applications [37]. The electrochemical performance of polypyrrole composites towards several compounds for electrochemical and gas sensors is also reported in several review papers [38,39,40]. Furthermore, the entrapment of several enzymes in conducting polypyrrole films to design biosensor devices is also reported in the bibliography [41,42,43].

Based on the previous subsections, the feasible employment of these conducting polymers in complex and high demanding sensing devices, such as electronic tongues (E-tongues) and noses (E-noses), is evident.

### 1.4. Electronic Systems: Electronic Tongues and Noses

**Figure 3:**
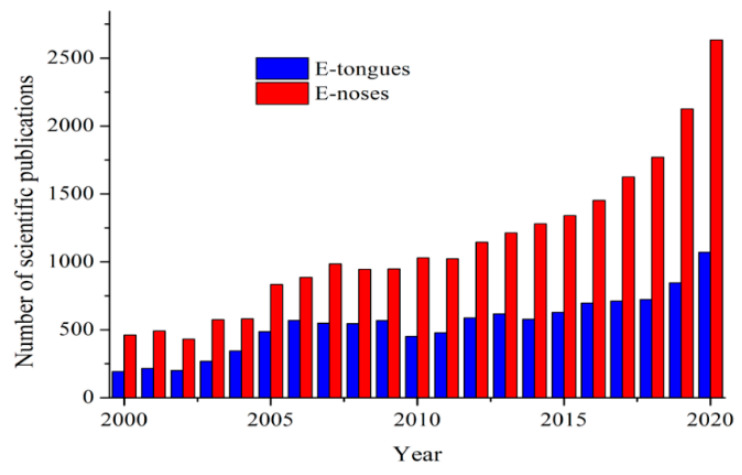
Number of scientific publications containing “electronic tongues” (E-tongues) and “electronic noses” (E-noses) terms published per year. Information obtained from Science Direct database (2021).

Electronic systems (E-tongues/noses) emerged during the beginning of the 21st Century as useful low-time consuming tools to obtain qualitative and quantitative information about several biological, pharmaceutical, and food samples. Currently, the demand for these devices has been largely increasing in the last years. The terms electronic “tongue” or “nose” arose due to their mimicking properties of taste and smell senses, respectively. To illustrate their growing scientific interest, the number of reports related to their development published per year is shown in Figure 3.

E-tongues/noses are mainly constituted by two blocks: a sensing unit, able to produce signals from target chemical species, and their processing part usually based on multivariate calibration methods. The latter permits the discrimination of samples, control of chemical adulteration (qualitative analysis), and correlation between experimental results and chemical parameters (quantitative analysis) by monitoring several features of the target samples [44,45,46,47,48].

#### 1.4.1. Sensing Unit: Electrochemical Sensor Arrays

Generally, several sensors are deployed as sensor arrays (sensing unit) in E-tongues and E-noses. The use of CPs coatings in the preparation of these sensors is strongly recommended due to their tailored electrochemical properties, reached by electronic doping, as well as their nanostructured morphology and thickness-controlled surface, as it was previously mentioned at the beginning of this section.

#### 1.4.2. Processing of the Collected Data: Multivariate Methods

Responses collected from unspecific sensor matrices are classified in specific response patterns and subsequently processed by chemometric analysis. The main multivariate techniques employed in the data analysis are principal component analysis (PCA) and partial least squares regression (PLS). PCA tries to reduce the number of highly correlated variables, converting them into uncorrelated variables which contain as much information as possible of the large data set (namely, principal components). By applying this procedure, samples are organized in groups, known as clusters, allowing their distinction according to different features. PLS intends to establish a correlation between independent and dependent variables. Thus, predictive models can be built, obtaining useful information [49].

#### 1.4.3. Novelty of the Work

Several pieces of research previously revised the application of conducting polymers and different conducting composites with nanomaterials, e.g., carbon nanomaterials, noble metal, and metal oxide nanoparticles, electrochemical sensors, biosensors, gas sensors, and immunosensors, among others [50,51,52,53,54]. In this review, the principal aspects concerning the development of E-tongues and E-noses based on CPs will be addressed. Firstly, the sensing units of both electrochemical sensors and chemiresistors will be explained, highlighting their composition. Secondly, the application of the resulting conducting polymer-based devices in the qualitative and quantitative analysis of the target samples by means of specific chemometric tools will be overviewed as well. Moreover, their performance will be discussed and related to characteristic features, such as surface morphology, thickness, and electrical conductivity. Finally, future trends of E-tongues and E-noses based on CPs and hybrid composite materials will be carefully analyzed and discussed.

## 2. Electronic Tongues (E-Tongues) Based on CPs

### 2.1. Sensing Unit: Electrochemical Sensors

**Figure 4:**
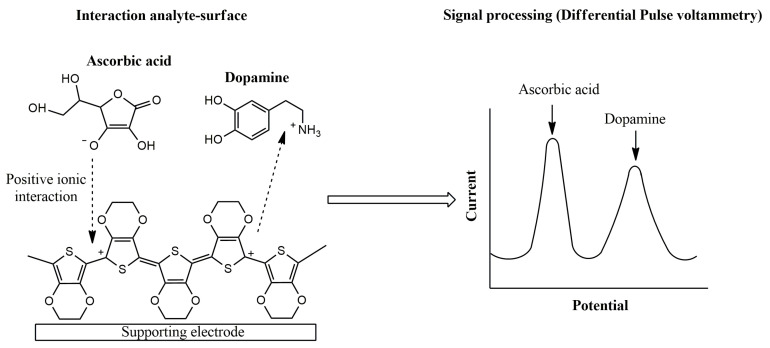
Proposed mechanism for ascorbic acid and dopamine interaction using PEDOT-modified electrodes.

The sensing mechanism of conducting polymer-based electrochemical devices has been deeply studied in different pieces of research [55,56]. As an illustrative example, the electrochemical oxidation of ascorbic acid and dopamine using PEDOT-modified sensors in a neutral medium was evaluated. Attractive ionic forces between ascorbate, usually found at neutral medium, and the conducting layer was proposed, leading to an electrocatalytic effect for ascorbic acid oxidation. On the other hand, a repulsive interaction between dopamine and the p-doped conducting film was established [57]. Therefore, the PEDOT sensor allows the simultaneous voltammetric determination of both analytes in neutral medium (Figure 4).

The interaction between the polymeric backbone and the target analyte was also reported for other heterocyclic conducting polymers, such as 3-amino-5-mercapto-1,2,4-triazole [58,59], polyaniline [60], and poly-(N-dimethylaniline) [61].

In addition to their doping properties, the inclusion of redox mediators in the conducting film may improve the electrochemical performance of the resulting devices for sensing diverse analytes of interest. In this regard, several electrochemical sensors employed in electrochemical assays in buffer and real matrices are listed in Table 1.

As it can be observed in the previous table, conducting polymers are very versatile in the determination of a wide variety of samples, including beverages (milk, wine, beer, juice, and water), pharmaceutical tablets (rutin and dopamine), and human body fluids (urine, blood, tears, and serum). Importantly, their analytical parameters, in terms of limit of detection and linear range, are competitive in comparison with other non-conducting polymer electrochemical sensors reported in literature, such as ceramic carbon and carbon paste modified sensors [90,91,92].

Furthermore, several analytes can be detected and quantified using conducting polymer-based electrochemical devices, all at once by single measurement. For example, PEDOT-modified materials were employed in the electrochemical sensing of ascorbic acid, uric acid, and dopamine simultaneously in biological samples, suggesting an electrocatalytic effect caused by the high ionic affinity between ascorbate anions and the conducting film at neutral media, as previously discussed in this subsection [93]. Other electrochemical sensors used for simultaneous determination purposes recently reported in bibliography can be also stated. In this sense, a PTh derivative was used in the electrochemical assays of three nitrophenol isomers in buffer and water media [94]. The modification of PANI film with a metal ion allowed the analysis of a quaternary mixture containing ascorbic acid, dopamine, uric acid, and tryptophan [95]. PANI-modified sensors can also detect simultaneously catechol, hydroquinone and resorcinol [96], lead and cadmium [97], and dopamine and uric acid [98]. PPy hydrogels and PPy-modified hydrogels are also highlighted for electroanalytical simultaneous sensing, as widely discussed in literature [99,100].

Not only can materials based on electrodeposited conducting polymer layers be employed as sensor transducers, but bulk material composites based on conducting polymers were reported in the bibliography as alternative electrochemical devices as well. For example, a carbon paste and PANI nanocomposite was employed for analytical sensing, suggesting a synergistic effect between starch, PANI, and carbon nanotubes [101,102]. Additionally, ceramic carbon-conducting polymer materials were also developed by means of sol-gel technology assisted by high-energy ultrasound. Resulting devices displayed remarkable analytical performance in the quantification of major phytocannabinoids, ascorbic acid, and 4-chloro-3-methylphenol. Furthermore, their surface can be renewed easily and quickly using either electrochemical or mechanical procedures [103,104]. Thus, similar bulk composite devices are promising materials for sensor arrays in electronic tongues, as discussed in the next subsection.

### 2.2. Analytical Application of E-Tongues

Electronic tongues have been successfully applied in the analysis of a wide range of samples. Their multiple applications in different ambits employing potentiometric and/or voltammetric sensor arrays were overviewed by many researchers [105,106,107,108].

The preliminary studies regarding electronic tongues containing conducting polymers developed by C. Mattoso and coworkers involves the use of ultrathin layers of PPy electrodeposited and their application in the distinction of some beverages [109,110,111]. One year later, De Saja developed an E-tongue by using PPy, PTh, and PANI coatings as voltammetric sensor arrays. Each one provided characteristic voltammetric signals, increasing the cross-selectivity of the resulting device and discriminating solutions with different tasting properties [112]. Moreover, conducting polymers were tested as well for qualitative analysis of tea and coffee samples and the quantitative determination of specific analytes contained in green Korean tea [113]. Notably, subsequent sensor arrays composed by conducting electrodeposited polymer coatings are relevant for food analysis at industrial scale. Table 2 shows several electronic tongues employed in electroanalysis of some foodstuffs.

**Figure 5:**
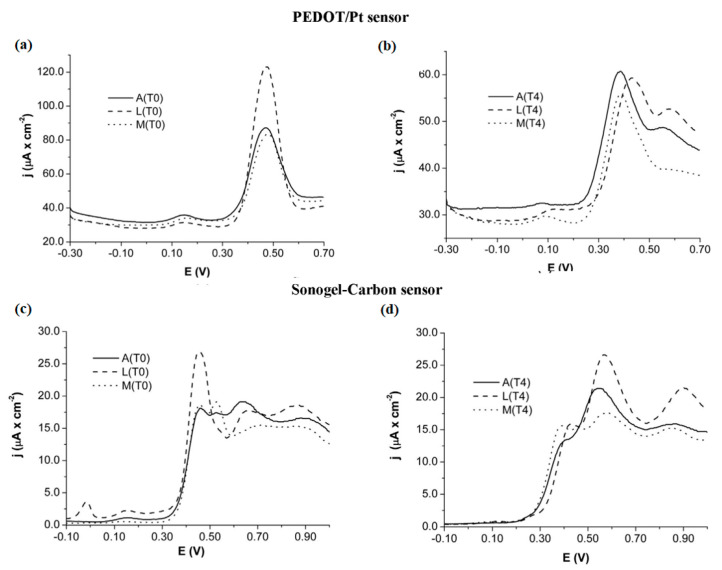
Voltammetric responses obtained with PEDOT/Pt (**a**,**b**) and sonogel-carbon sensors (**c**,**d**) for three types of Italian grape varieties, Ancellota (A), Lambrusco Marani (L) and Malbo Gentile (M), collected at 10 days (T0) and 50 days (T4). Reprinted with permission from ref. [114]. Copyright 2018, Elsevier.

A cursory inspection of Table 2 exposes the high versatility of electronic tongues for beverage analyses, such as wine, beer, and juices. Regarding the analysis of wines and musts, Pigani et al. proposed an interesting application to assess the influence of ripening time. Voltammetric responses displayed with PEDOT and sonogel-carbon electrodes for must samples collected after 10 and 50 days of ripening (namely T0 and T4, respectively), were remarkably different, as is shown in Figure 5. Thus, the use of voltammetric sensors in this work to provide useful information about the ripening process, in addition to other relevant parameters studied by chemometric analysis, such as pH, total acidity, total sugars, and anthocyanins [114]. Furthermore, the shape and scan evolution of voltammetric signals are actually informative with respect to the discrimination of samples analyzed, as is detailed in literature [115]. Likewise, PPy films deposited with different doping agents were also reported as sensor arrays of E-tongue systems applied to red wines.

**Figure 6:**
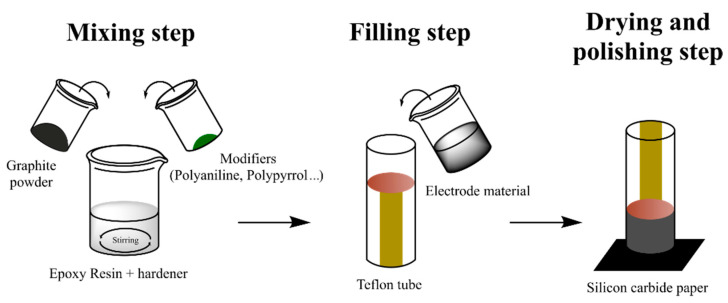
Fabrication of epoxy-resin electrodes modified with CPs powders.

Chemometric analysis was performed in all cases, leading to relevant conclusions. Briefly, PCA analysis allowed the clear differentiation of wines with different SO_2_ content. Considering that SO_2_ should be ranged in a specific concentration interval, samples with outlier values can be identified and, therefore, the screening of chemical adulteration in red wines can be successfully assessed [121]. Differently, Arrieta et al. studied redox processes of different alcoholic beverage samples (wine and beer). The authors employed PPy based sensor arrays to build a model able to predict α-isoacid and alcoholic degree content by PLS method. In addition, dark beers were discriminated from pale beers and alcohol-free samples considering PCA score plots [123]. Alternately, Garcia-Hernandez et al. were also able to differentiate wine samples according to the polyphenol content studied by PANI coated sensors by means of PCA analysis. Interestingly, the analytical methodology reported in this work combines the use of the electronic tongue and the infrared spectroscopy to estimate fourteen chemical parameters of red wines in a few minutes [119]. It is noteworthy to mention the use of hybrid composites constituted by conducting polymer powders instead of coatings in electronic tongues by M. del Valle and coworkers. Graphite-epoxy resins modified with metallic, cobalt, PANI, and PPy powders were employed as voltammetric sensor arrays in qualitative and quantitative analyses of wines [126]. Figure 6 depicts the usual fabrication of epoxy-resin electrodes modified with CPs powders.

**Figure 7:**
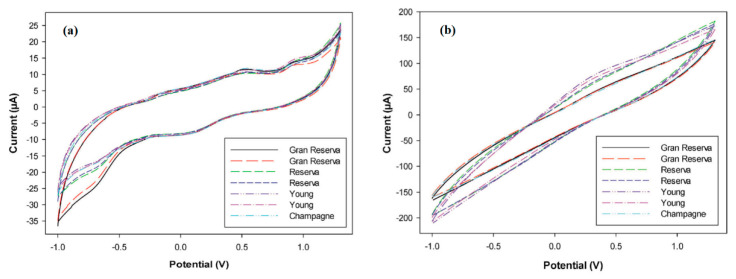
Cyclic voltammograms recorded with (**a**) PPy–graphite epoxy and (**b**) PANI–graphite epoxy sensors for different wine and cava samples. Adapted with permission from ref. [127]. Copyright 2014, John Wiley and Sons.

The resulting electronic tongue was able to distinguish several types of wines [126] and cavas [127] (Figure 7), detecting undesired products from their elaboration, as well as to determine the polyphenol index of a large number of wine samples [128]. In the last report, the identification of some polyphenolic compounds using a PPy-modified sensor was also performed, opening up the possibility to quantify individual polyphenols present in a complex mixture—an issue of paramount importance for the successful assessment of antioxidant capacity—with a simple and low-time consuming analytical tool.

Despite the wide application of E-tongues to alcoholic beverages, other food samples have already been studied by these devices as well. For example, another PANI-based tongue mixed with polyamide was capable to discriminate several bovine milk samples based on their tetracycline content by using PCA analysis [117]. Furthermore, the residue concentration can be clearly distinguished. A polymer sulfanilic acid film (PBSA) was recently integrated in an E-tongue system capable to discriminate rice wines from different local origins targeting ascorbic acid, glucose, and tyrosine [129,130]. Additionally, poly-(alkoxy-bithiophenes) and polylactic acid based sensors were employed in the discrimination of diverse taste solutions [131,132].

To a lesser extent, the employment of electronic tongues based on conducting polymer coatings in environmental monitoring has also been reported. One illustrative example is the research piece of Braga et al. In this work, water samples were classified according to the 2-methylisoborneol and geosmin contents, two toxic substances derived from algae decomposition, using sensor arrays constituted by PANI layers. Moreover, the concentration of both analytes could be monitored at values as low as 25 ng/L in tap and distilled water [133]. Similarly, Carvalho et al. employed PANI coatings as working electrodes in combination with poly(o-ethoxyaniline), sulfonate lignin, and aquatic humic substances to analyze tap water. The resulting electronic tongue was able to discriminate water samples collected from diverse locations on the basis of different psychochemical parameters, such as pH, temperature, and turbidity, among others [134]. On the other hand, Facure et al. proposed sensors arrays based on PPy and PEDOT/PSS in combination with rGO for the discrimination of two commercial pesticides, Malathion and Cadusafos. Based on PCA results reported in this work, both analytes can be distinguished at nanomolar level in buffer and tap water samples [135]. Poly(o-ethoxyaniline) and nylon 6 were used as sensing units in the determination of paraoxon in corn top. The resulting electronic tongue was capable to discern paraoxon contamination in water samples [136].

As it was mentioned in Section 1, enzymes can be immobilized into the conducting polymer coatings, enhancing the selectivity of the device. This approach has been reported in several research works, such as the incorporation of tyrosinase by the electrodeposition of PEDOT onto a sonogel-carbon surface [72] and the simultaneous electrodeposition of PPy and tyrosinase on platinum [137,138]. Particularly, C. Garcia-Hernandez and coworkers developed a bio-electronic tongue containing both tyrosinase and glucose oxidase enzymes with PPy films doped with gold nanoparticles collectively deposited onto platinum and stainless-steel substrates. The resulting devices were able to predict total polyphenol index and sugars of grapes using chemometric analysis based on the specificity provided by the biological recognition elements towards polyphenols and sugars. Additionally, the polyphenol index and the alcoholic degree of wines were also predicted [139].

The effect of nanostructured surface morphology on the sensing behavior should also be considered. For instance, PEDOT nanorods were deposited on a glassy carbon surface and applied for electrochemical detection of nitrite, exhibiting high sensitivity due to the good dispersibility and large surface area [140]. Other work reported the deposition of PEDOT nanorods and graphene oxide sheets (GO) on glassy carbon. The resulting electrochemical sensor displayed the best sensitivity with PEDOT/GO for rutin detection compared with the bare device or those modified with PEDOT or GO. This result can be attributed to the high surface area, which provides a high number of active sites to increase the electron transfer between the analyte and electrode [141]. Another work studied the influence of film thickness on the electrochemical performance using interdigitated microelectrodes based on poly(o-ethoxyaniline). The sensitivity was increased with the thickness of the film, which can be ascribed to higher roughness morphology [142]. Another scientific work reported the development of cobalt oxide nanosheets embedded with PANI nanofibers and employed as biosensors for glucose oxidation. The thickness of the conducting film on cobalt nanosheets plays a relevant role: an increased thickness of PANI resulted in a long diffusion layer between the electrolyte and the surface, making the analyte/surface electron transfer difficult. On the other hand, lesser film thickness may contain a low number of active sites, leading to lower oxidation current values. For these reasons, the thickness of PANI was optimized with the aim to obtain an electrochemical device with a remarkable electrochemical performance [77].

## 3. Electronic Noses (E-Noses) Based on CPs

### 3.1. Sensing Unit: Chemiresistors

In principle, three types of sensing units can be employed in gas sensing: chemiresistors, quartz crystal microbalance gravimetry, and optical sensors [143]. Among all of them, the first one is, by far, the most employed unit in electronic noses, and thus, this review will be focused on chemiresistors as the sensing unit.

The sensing performance of chemiresistor-based conducting polymers has been widely reported in bibliography. In brief, after exposing the conducting film to gases, the resistance changes depending on the initial concentration of the flowing gas. The overall resistance (S) measured with the p-doped polymeric device was calculated by the ratio between the resistance in air (R_a_) and the resistance in the presence of the flowing gas (R_g_) by using the following equation [144].

(1) $$S=\frac{\left|R_{g}-R_{a}\right|}{R_{a}}\times 100$$

**Figure 8:**
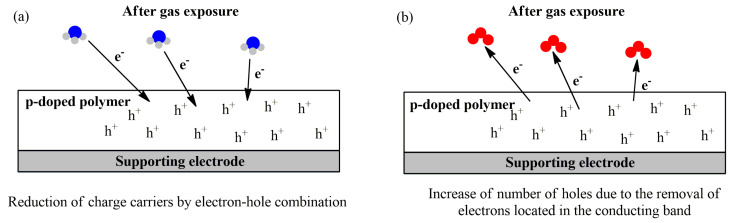
Overall mechanism of gas sensing using a reducing gas (NH_3_) (**a**) and an oxidant gas (O_3_) (**b**) as examples.

The sensing mechanism of p-doped conducting polymers towards different pollutants is overviewed in several review papers [145,146,147,148] and shown in Figure 8. The target gas can act as an electron donor of the conducting polymer layer, leading to a decrease in the number of holes by electron-hole combination, and thus, increase the charge resistance. On the other hand, electrons from the conducting band of the polymer can be removed by an electron-acceptor compound, leading to the increase in the number of holes, and, hence, decrease the electrical resistance.

The p-doped polymer resistance changes depending on the nature of the target analyte: oxidant gases, such as NO_2_ and O_3,_ induce an increase in the number of major charge carriers, decreasing the resistance (Figure 8b). Reducing gases, such as NH_3_, CO, and H_2_S, induce the opposite effect by decreasing the charge carriers of the conducting film (Figure 8a) [149].

**Figure 9:**
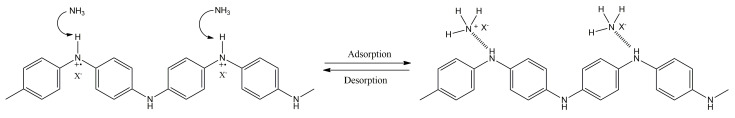
Possible interaction mechanism between ammonia and PANI.

The protonation/deprotonation of the conducting layer due to the vapor exposure is also reported in the literature for some conducting polymers, such as PANI [150,151,152]. Figure 9 illustrates a possible interaction between ammonia, a reducing gas widely employed as model analyte, and PANI.

As it can be observed in the previous figure, PANI can be deprotonated under ammonia exposure, leading to the de-doped state of PANI. This process is reversible, and thus, PANI can be protonated again, leading to the desorption of ammonia. The performance of some chemiresistors based on conducting polymers, in terms of concentration detected, response and recovery times, are shown in Table 3.

Upon scrutiny of Table 3, it is possible to note that several volatile compounds can be detected using gas sensors comprised by conducting polymers. Remarkably, low recovery and response times were obtained in all cases, as well as negligible influence of humidity in some cases, indicating good sensing performance [161]. Another example regarding the employment of PANI-based flexible devices for the gas sensing of several volatile biomarkers can be found in the work published by Deng et al. [166]. In this work, high tolerance of this sensor to the humidity at room temperature was reported. Therefore, it becomes apparent that the employment of conducting polymers to constitute electronic noses for volatile compounds detection is feasible and highly recommended.

### 3.2. Analytical Application of E-Noses

Among the multiple possible applications of E-noses, the early diagnosis of diseases and the evaluation of food quality in a non-invasive manner are the most relevant for society. Importantly, electronic nose devices based on conducting nanocomposites have proved their suitability in both scenarios during the last decade [167,168,169,170].

Despite its high interest currently, investigations of the role of conducting polymers in electronic noses started in the previous century, with the development of PPy, PTh, and PANI derivatives to detect alcoholic volatile compounds [171]. However, new discoveries have been carried out at the beginning of this century, leading to great improvements in the development of these devices. In this regard, Stella and coworkers developed an E-nose system based on PEDOT, PANI, and PPy coatings for the distinction of three Italian olive oils by using their aromatic substances content as a differentiating parameter [172]. Contrarily, other authors have vastly explored the role of several dopants. For example, Barisci et al. developed gold tracks supported on silicon chip coated with PPy doped with 12 different chemical compounds to detect aromatic hydrocarbons, benzene, toluene, ethylbenzene, and xylene [173]. In spite of the lack of concise explanations, the authors must be praised for the wide spectrum of polymers assayed. In fact, the evaluation of different dopants in CPs seems to be the quintessence of a great number of pieces of research. Particularly, PANI coatings with different doping agents are commonly reported in bibliography as sensor arrays in E-nose devices to monitor several parameters in foodstuffs and human body fluids [174,175,176]. Table 4 shows some illustrative examples recently reported in the literature.

The electrochemical performance of electronic noses based on PANI is reported in the previous table. As observed, there is a vast exploitation of dopant agent employment for the preparation of PANI sensors in all ambits. The research performed by Tiggemann et al. in this field proposing a PANI film doped with CSA deposited on PGIE (PANI-CSA/PGIE) should be praised. The resulting film was extensively characterized by SEM and proved that it led to faster response and high sensitivity for strawberry and apple in comparison with PANI-HCl/PGIE and PANI-DBSA/PGIE, with less porous and homogeneous surfaces. Interestingly, it was also demonstrated that porous morphology allows the flowing of the target gas into the substrate, facilitating gas diffusion and hence, increasing the detection rate [181]. Additionally, it was stated that the interaction between the volatile compound and the conducting polymer surface also plays an important role in the sensitivity displayed with the sensor. In the same report, higher sensitivity for grape aromas was achieved with PANI-HCl/PGIE film compared with those obtained with PANI-CSA/PGIE and PANI-DBSA/PGIE under the same conditions. This can be justified based on a favorable interaction between PANI-HCl/PGIE surface, homogeneous and regular, and volatiles with bulky groups, such as methyl anthranilate ester, commonly found in grape aromas. Other authors have drawn similar conclusions. For example, the higher resistance variation found with the PANI/chitosan sensor reported by Maout and coworkers can be also explained in terms of coating morphology. In this case, PANI clusters were embedded into the insulating chitosan matrix at different depths, providing higher resistive responses [182]. Furthermore, the morphology of carbon allotrope/PANI surface was also studied for gas sensing of different essential oils. The rougher morphology appreciated in PANI/GO surfaces led to faster interaction with volatile organic compounds (VOCs) with respect to PANI/MWCNT surfaces [184].

Despite the wide use of PANI films in E-noses, the employment of PTh derivatives and PPy coatings in E-nose arrays should be stressed as well. In this regard, discrimination of some VOCs was carried out using an E-nose system based on hybrid PEDOT/graphene films arrays. Tung et al. reported a dual doping role of VOCs: by controlling the carrier mobility in graphene layers and by inducing a conformational change of PEDOT chains. Moreover, a synergistic effect between graphene and PEDOT was described in this work, yielding high electrical performance [185]. Other work reported the employment of PPy coatings doped with several counter anions in the discrimination of VOCs and the sensing of several alcoholic compounds [186,187], highlighting the role of the morphology of polymer coating in the responses collected from each sensor. Alternatively, poly (3-hexylthiophene) and poly(9,9-n-octyl-2,7-fluorenylenevylene-alt-4,7-dibenzothiadiazole-2,5-thiophene) (PF-TBT) were used in E-nose for the detection of several gases, used as biomarkers for diseases and environmental monitoring [188] and for the discernment of tobacco samples, in combination with diverse porphyrins [189], respectively. Another polyvinyl derivative, (poly[2-methoxy-5-(2- ethylhexyloxy)-1,4-phenylenevinylene)] (MEH-PPV), was used as the active layer with a porous silicon structure for NO_2_ gas sensing. Based on its promising analytical performance, it can be applied as an E-nose for environmental monitoring [190]. A recent work published by Jafari and Amini reported a PPy electrochemical device as a promising sensing unit for an E-nose system to detect lactic acid gas [191].

On the other hand, Rañola et al. have focused their efforts on research involving CPs composite materials in the analysis of oil samples. In this case, poly(3-methylthiophene) was employed together with PANI and PPy sensors to discriminate different virgin coconut oil samples. After using PCA analysis, rancid coconut oil could be easily distinguished from refined ones [192].

Other foodstuffs can be analyzed using E-nose devices based on CP composites. An E-nose containing several ethylene and vinylene derivatives was employed in the evaluation of the biodeterioration of oranges by fungus species. Differences between oranges after the first day of incubation and those non-inoculated (control oranges) were established by means of PCA analysis. Interesting findings were obtained with the device, since the color of oranges slightly changed after the first day of incubation compared with the control group; thus, visual differences between both groups were not found, in contrast with significant differences obtained with the E-nose system [193].

Even if E-noses are more devoted to foodstuff or environmental analysis, there are some researchers that try to promote them in the health sector as well. An interesting piece of research was exhibited by Castro and coworkers reporting an E-nose based on different polymer matrices and MWCNTs as a lung cancer biomarker detector for the discrimination of several organic vapor solvents (toluene, methanol, ethanol, and water vapor, among others) using PCA analysis [194]. Additionally, other authors have proposed a carbon transducer based on poly (ether-imide) as a cancer biomarker for the detection of several solvents. In this work, it was demonstrated that the aromatic bulky pendant groups in the polymeric layer established strong attractions with the CNT surfaces, which allow the creation of active sites where analyte molecules are adsorbed, improving the sensitivity of the device [195].

As in the case of electronic tongues based on conducting polymers, the presence of nanostructures can improve the performance of E-noses. A critical review regarding the employment of nanostructured materials for gas sensing was offered in the previous decade [196]. A chemiresistor constituted by a PEDOT nanocomposite exhibited excellent sensitivity and selectivity for NO_2_ detection, which can be explained by the enhancing of gas sorption/desorption due to its porous nanostructured surface [197]. PPy nanoparticles were employed for sensing of ammonia and methanol vapor gases. Regardless of the analyte, sensitivity was increased at lower particle size of PPy. Furthermore, the device composed of PPy nanoparticles provided low response and recovery times [198]. Other examples of the influence of nanostructures for gas sensing can be found in the work published by Ma and coworkers. In this work, porous nanostructures of PANI allow a sensitive detection of amines, proposing an extensive gas sensing mechanism [199]. Another work reported the sensing of acetone with a poly-(3-hexylthiophene) nanofibers sensor [200]. Fast rise time was obtained with this device, which can be ascribed to the high number of sites for gas/material interaction available to the analyte along the nanofiber.

## 4. Future Perspectives: Integration of E-Tongues and E-Noses in Commercial Systems

It is not ambitious to think that the analytical applications of E-tongues/noses possess a great impact, not only in the foodstuff ambit but also in the health and environmental sector. Furthermore, this impact is rising sharply, reflecting the great need in society for these devices. Therefore, their implementation in commercial devices is exceedingly pursued by many sensor companies. Currently, there are some examples of its commercialization.

### 4.1. Commercial Prototypes of E-Tongues

Concerning E-tongues, Alpha M.O.S (Tolouse, France) and Insent Inc (Kanagawa, Japan) offer two models (αAstree and TS-5000Z, respectively) that have been used in the evaluation of food quality in the last decade [201,202,203,204,205]. Other laboratory prototypes were also employed for pharmaceutical analysis, providing very satisfactory results like those obtained with commercial systems [206].

### 4.2. Commercial Prototypes of E-Noses

Regarding E-noses, a commercial system containing several conducting polymers as sensor arrays (Cyranose 320^®^), offered by Sensigent (California, USA), was employed in the screening of several diseases (breast and lung cancer [207,208,209], asthma [210,211], and amyotrophic lateral sclerosis [212], among others), identification of foodstuffs (rice, wines [213], and fruits [214]) and classification of road asphalt samples [215,216]. Additionally, fecal VOCs can be inspected as well, informing about the microbial enterotype of infants [217]. Other companies also supply E-noses. For example, AromaScan A32S^®^ (Osmetech Inc. London, UK) provides useful information about the diagnosis of urban trees, being able to discriminate VOCs from healthy and decaying woody samples [218], and the assessment of the quality of catfish meat [219]. In this work, off-flavor in catfish filets can be identified from good-flavor ones by means of PCA. Notably, the new device tested displayed promising features for the analysis of commercial beverages [220].

### 4.3. Final Remarks: Challenges of Electrochemical/Gas Sensing Devices

Despite the excellent analytical results provided at laboratory scale in food, pharmaceutical, and medical sectors, only some timid examples can be found commercially available. In our modest opinion, the inclusion of CPs and their development may pave the way to keep growing and reach the desired applicability of E-tongue and E-nose systems. Currently, in order to reach higher technological readiness levels (TRLs), the developed devices must be able to perform reliable, robust, fast, accurate, and in-situ measurements using diverse samples, by using a non-complex, low cost, and portable instrumentation. The stability of the conducting coatings is another issue to take into account, since the repeatability of the responses provided with the devices can be affected. The conducting film may be passivated after performing successive electrochemical assays, and film overoxidation can take place at high potentials as well. Furthermore, stability can be affected by swelling/deswelling phenomena. With the aim to minimize these factors, several parameters, including analyte concentration, film characteristics (e.g., thickness and morphology) and instrumental conditions should be carefully controlled. Further research in this sense is under study to accomplish all the commercial requirements mentioned.

## 5. Conclusions

The analytical utility of E-tongues and E-noses containing intrinsic conducting polymers for analytical monitoring purposes has been briefly overviewed. E-tongues demonstrate their usefulness in important areas of food industry, such as chemical adulteration, classification of foodstuffs, determination of polyphenol indexes, bitterness evaluation, etc. The use of voltammetric sensor arrays and their combination with spectroscopic techniques provide useful information of the sample, as can be found in some works collected in this review. Other sectors can be encompassed with these devices, i.e., environmental monitoring. Hybrid conducting polymer composites based on epoxy resins can be also used as sensor arrays, displaying several advantages, such as renewable electrode surface and stability in different solvents. Contrarily, E-tongues can be also constituted by enzymatic sensors, which provides selectivity for the analysis of sugars and polyphenols, among others. On the other hand, E-noses are employed in foods and body fluids, being useful in food quality and diagnosis of several diseases. The electrochemical performance of the CP sensor arrays is affected by the conducting coating morphology, as was previously discussed. The interaction between polymer electrode surface and target volatile compounds also plays a key factor in the analytical performance of the E-nose. Based on the above, conducting polymer based-electronic systems are promising for analytical purposes in the in-situ screening of several adulterations and diseases, providing reliable and accurate results extremely fast. Major attention in the development of commercial electronic tongues and noses will be focused on future investigations, allowing in situ monitoring of several features of target samples.

## Figures and Tables

**Table 1:** Electrochemical sensors based on polythiophene (PTh), poly-(3,4-ethylenedioxythiophene) (PEDOT), polyaniline (PANI), and polypyrrole (PPy) currently reported in literature.

Electrochemical Device	Analyte	Working Media	Sample	Analytical Parameters		Ref.
				LD (µM)	LR (µM)	
PTh						
MWCNT/PTh/Pt	BPA	PBS pH 7.5	Water	0.009	0.05–0.4	[62]
MnO_2_/PTh/rGO/GCE	MP	PBS pH 7	Human urine and blood	0.0057	0.5–10	[63]
GO-4-ATP-Au-PTh/Au GCE	Nicotine	PBS pH 7	Serum, urine, cigarette	0.17	1.0–30	[64]
PTh-AgBr	Glucose	NaOH	Human blood plasma	0.31	4–5000	[65]
PTh-Ag/GCE	L-Tryp	PBS pH 7	Soybeans extract	0.020	0.2–400	[66]
PEDOT						
PEDOT/IL/GCE	DA	PBS pH 7.4	Human urine	0.033	0.2–328	[67]
UiO-66-NH_2_@PEDOT/GA/GCE	PCMC	ABS pH 6	Tap water	0.2	0.6–18	[68]
PEDOT/AG/GCE	AC	PBS pH 7	Local tablets	0.041	0.15–5881	[69]
Cu_2_O/PEDOT/MWCNT	Glucose	NaOH	Human blood serum	0.04	0.495–374	[70]
GC/PEDOT-AuNPs-SV	CA	PBS pH 7	Juice	4.24	10–1000	[71]
PEDOT-Tyr/SNG-C	CA	PBS pH 7	Wine, beer	4.33	10–300	[72]
PEDOT/PEDOT-SH/Au	Nitrite	PBS pH 6.9	Tap water, milk	0.051	0.15–1000	[73]
PEDOT/Au	UA	PBS pH 6.6	Milk	7.0	6–200	[74]
GCE/PEDOT-MC/AgNPs	Rutin	PBS pH 3	Tablets	0.0035	0.005–0.5	[75]
Pt/PEDOT-PBNPS	H_2_O_2_	ABS pH 5.5	Human blood	1.4	5–1000	[76]
PANI						
Co_3_O_4_@PANINFs/GCE	Glucose	PBS pH 7.4	Human serum	60	100–8000	[77]
TiO_2_@PANI@Au/GCE	Hydrazine	NH_3_/NH_4_^+^ pH 9	Power plant sewage	0.15	0.9–1200	[78]
PANI/SnO_2_/GCE	Nitrite	PBS pH 6	-	0.04	0.12–7777	[79]
GCE/PANI-Fe_3_O_4_	DA	PBS pH 7	Water	0.176	0.2–2.4	[80]
GCE/PANI-NiO	DA	PBS pH 7	Water	0.166	0.2–2.4	[80]
α-Fe_2_O_3_/PANI/GCE	UA	PBS pH 7	Human urine	0.038	0.01–5	[81]
NiO-NPs@PANINS/SPE	Glucose	NaOH	Human blood serum	0.06	1–3000	[82]
MeGO/PANI	AA	PBS pH 7.4	-	2.0	8–5000	[83]
PPy						
Fe_3_O_4_@PPy/MWCNTs/GE	AT	BR pH 4	Serum, tablets	0.0230	0.0314–201	[84]
AuNP/PPy/GCE	L-dopa	PBS pH 7	Urine	0.075	0.1–6.0	[85]
PDA/PPy/GCE	UA	PBS pH 8	Human serum, urine	0.11	0.5–40	[86]
PGE/CuO-NPs/PPy	TR	PBS pH 8.5	Tablets	0.001	0.005–380	[87]
PPy:LAC	Lactate	KNO_3_	Human tear, rat blood	81.0	100–10,000	[88]
AuCu/PPy/Cu-TCCP	H_2_O_2_	PBS pH 8	Medical H_2_O_2_ solution	0.0067	0.71–24,100	[89]

AA: ascorbic acid; ABS: acetic buffer solution; AC: acetaminophen; AT: atorvastatin; ATP: adenosine triphosphate; BPA: bisphenol A; BR: Britton-Robinson; CA: caffeic acid; CuO-NPs: copper oxide nanoparticles; DA: dopamine; PTh: polythiophene; GA: graphene aerogel; GCE: glassy carbon electrode; IL: ionic liquid; LAC: lactate; LD: limit of detection; LR: linear range; L-Tryp: L-tryptophan; MC: mesoporous carbon; MP: methyl parathion; MWCNT: multi-walled carbon nanotubes; PANI: polyaniline; PANINS: polyaniline nanofibers; PBNPS: Prussian blue nanoparticles; PBS: phosphate buffer solution; PCMC: p-chloromethylcresol; PEDOT: poly-(3,4-ethylenedioxythiophene); PGE: pencil graphite electrode; PPy: polypyrrole; rGO: reduced-graphene oxide; SPE: screen-printed electrode; SV: sinusoidal voltage; TCCP: meso-tetra-(4-carboxyphenyl)-substituted porphyrins; TR: tramadol; and UA: uric acid.

**Table 2:** Electronic tongues containing sensors based on conducting polymer coatings applied in the analysis of food samples.

Sensor Array		Sample	Use	Multivariate Calibration	Ref.
No CP Sensor	CP Sensor				
SNG-C	PEDOT/Pt	Musts	Discrimination of samples collected at different ripening times	PCA iPLS PLS	[114]
-	PEDOT/Pt	Red wines	Classification of different samples and origin	PCA PLS	[115]
Pt Au	PEDOT/Pt	Fruit juice	Discrimination between samples from different fruits	PCA PLS-LDA	[116]
IDE PA6/IDE	PA6/PANI/IDE (0.25–5.0% PANI)	Bovine milk	Discrimination of samples according to tetracycline residue content	PCA	[117]
CE AuCE rGO-CE rGO-AuCE	PANI-CE PANI-AuCE	Vinegar, sugar	Multiflavor detection	PCA	[118]
C/SPE NiO/C/SPE MWCNT/C/SPE SWCNT/C/SPE Pt	PANI/C/SPE	Red wine	Phenolic content	PCA	[119]
SWCNT/SPCE MWCNT/SPCE	PPy-DSA/SPCE	White wine	Discrimination according to varietal origin	PCA LDA	[120]
CPE-CoPc CPE-LuPc_2_ CPE-LuPc_2_	PPy-dopant/Au Dopant: SO_4_, DSA, FCN, AQDS, PWA, TSA	Red wine	Evaluation of chemical adulteration	PCA PLS	[121]
GdPc_2_/CSPE DyPc_2_/CSPE CSPE	PPy-dopant/CSPE Dopant: FeCN, NP, Mo	Beef	Determination of ammonia and putresceine	PCA PLS-LDA	[122]
-	PPy- dopant/Pt Dopant: DSA, H_2_SO_4_, FCN, AQDS, PWA, TSA	Beer	Evaluation of bitterness and alcoholic strength	PCA PLS	[123]
-	PPy-dopant/Pt Dopant: FCN, NP, PWA, H_2_SO_4_, MO, AQS	Olive oil	Evaluation of bitterness	PCA PLS	[124]
-	PPy-dopant/SPCE Dopant: DSA, SO_4_, FCN	Wine	Classification of wines according to vintage year	PCA LDA	[125]
Graphite-epoxy PtNPs CuNPs	PANI PPy	Wine	Classification of wines and recognition of the oxygenation effect	PCA	[126]

AQDS: anthraquinone-2,6-disulfonic acid, disodium salt; AQS: anthraquinone-2,6-disulfonic acid; CNT: carbon nanotubes; CoPc: cobalt phthalocyanine; CPE: carbon paste electrode; CuNPs: copper nanoparticles; DSA: sodium 1-decanesulfonate; FCN: potassium hexacyanoferrate (II); IDE: interdigitated electrodes; LDA: linear discriminant analysis; LuPc2: lutetium bis-phthalocyanine; MO: sodium molybdate; MWCNT: multi-walled carbon nanotubes; PA6: polyacrilamide; PANI: polyaniline; PCA: principal component analysis; PEDOT: poly-(3,4-ethylenedioxythiophene); PLS: partial least squares regression; PPy: polypyrrole; PWA: phosphotungstic acid; PtNPS: platinum nanoparticles; rGO: reduced-graphene oxide; SNG-C: sonogel-carbon; SPCE: screen-printed-carbon electrode; SPE: screen-printed electrode; SWCNT: single-walled carbon nanotubes; and TSA: p-toluenesulfonic acid.

**Table 3:** Chemiresistors based on polythiophene (PTh), poly-(3,4-ethylenedioxythiophene) (PEDOT), polyaniline (PANI), and polypyrrole (PPy).

Gas Sensor Device	Target Gas	Range (ppm)	Sensing Performance			Ref.
			Gas Conc. (ppm)	Recovery Time (s)	Response Time (s)	
SnO_2_/PTh	NO_2_	10–200	10	-	2.07	[153]
P3CT/CNT	NMPEA	0.004–0.032	0.004	40	20	[154]
PEDOT:PSS/FeCl_3_	NH_3_	0.2–200	0.5	-	20	[155]
WO_3_-PEDOT:PSS	LPG	500–3000	500	54	29.4	[156]
PANI/PVDF	NH_3_	0.2–5	0.2	235	174	[157]
PANI/SnO_2_	NO_2_	5–55	37	25	17	[158]
SnO_2_/rGO/PANI	H_2_S	0.05–10	2	78	82	[159]
PANI-NF	LPG	100–1000	700	200	50	[160]
PPy/rGO	NH_3_	1.0–4.0	1.0	300	60	[161]
PPy thin film	NO_2_	10–100	10	374	218	[162]
PPy nanoribbons	CH_3_CH_2_OH	-	100	31	2	[163]
PPy-Ag	CH_3_COCH_3_	25–600	580	150	175	[164]
PPy-CNT	H_2_	1–100	10	-	>1.0	[165]

CNT: carbon nanotubes; LPG: liquified petroleum gas; NF: nickel ferrite; NMPEA: n-methylphenethylamine; P3CT: poly[3 -(6-carboxyhexyl)thiophene-2,5-diyl]; PANI: polyaniline; PEDOT: poly-(3,4-ethylenedioxythiophene); PPy: polypyrrole; PSS: poly(styrenesulfonate); PTh: polythiophene; PVDF: polyvinylidene; and r-GO: reduced-graphene oxide.

**Table 4:** Electronic noses based on polyaniline (PANI) films applied for analytical purposes in the last decade.

PANI Sensor Array	Sample	Use	Multivariate Calibration	Ref.
PANI-dopant/IDGEs Dopant: CSA, DBSA, HCl	Strawberry Grape Apple	Discrimination of samples according to aromatic substances	PCA	[177]
PANI-HCl/PGIEs PANI-HCl/IDEs	Strawberry Grape Apple	Detection of different aromas	PCA	[178]
PANI-dopant/IDGEs Dopant: HCl, TSA, CSA, MSA	Cow’s estrus	Determination of estrus times of cows	PCA	[179]
PANI-dopant/IDEs Dopant: HCl, TSA, CSA, MSA	Bananas	Monitoring of bananas ripeness	PCA	[180]
PANI-dopant/PGIEs Dopant: CSA, HCl, DBSA	Gummy candies	Monitoring of aromas during candy storage	PCA	[181]
PANI-CSA/Chitosan PANI-DBSA/TiO_2_ PANI-DBSA/CNT	Simulated human breath	Preliminary diagnoses of kidney disease	PCA LDA	[182]
PANI/AuNPs	Human breath	Early diagnoses of renal diseases	PCA LDA	[183]
PANI-dopant/MWCNT PANI-dopant/GO Dopant: CSA, DBSA, HCl	Essential oils	Determination of quality of essential oils	PCA	[184]

CSA: camphorsulfonic acid; DBSA: dodecylbenzenesulfonic acid; GO: graphene oxide; IDE: interdigitated electrode; MSA: methanesulfonic acid; MWCNT: multi-walled carbon nanotubes; PANI: polyaniline; and TSA: p-toluene sulfonic acid.

## Data Availability

Not applicable.
